# Elevated frequencies of CD14^+^HLA-DR^lo/neg^ MDSCs in COVID-19 patients

**DOI:** 10.18632/aging.202571

**Published:** 2021-02-26

**Authors:** GuoHui Xue, Mei Jiang, Rui Zhao, AiPing Le, JunMing Li

**Affiliations:** 1 Department of Clinical Laboratory, The First Affiliated Hospital of Nanchang University, Nanchang 330006, Jiangxi, China; 2 Department of Transfusion, The First Affiliated Hospital of Nanchang University, Nanchang 330006, Jiangxi, China

**Keywords:** SARS-CoV-2, COVID-19, immunosuppression, MDSC

## Abstract

Background: The immune responses, hyper-inflammation or immunosuppression, may be closely related to COVID-19 progression. We aimed to evaluate the changes of frequency of CD14^+^HLA-DR^lo/neg^ MDSCs, a population of cells with potent immunosuppressive capacity, in COVID-19 patients.

Methods: The levels of CD14^+^HLA-DR^lo/neg^ MDSCs were determined by flow cytometry in 27 COVID-19 patients, and their association with clinical characteristics and laboratory data were analyzed.

Results: The frequency of CD14^+^HLA-DR^lo/neg^ MDSCs was elevated in COVID-19 patients, particularly severe patients. A follow-up comparison revealed a decline of CD14^+^HLA-DR^lo/neg^ MDSCs percentages in most patients 1 day after testing negative for SARS-CoV-2 nucleic acid, but the levels of CD14^+^HLA-DR^lo/neg^ MDSCs were still greater than 50.0% in 3 ICU patients 4-10 days after negative SARS-CoV-2 results. Elevated frequency of CD14^+^HLA-DR^lo/neg^ MDSCs was positively correlated with oropharyngeal viral loads and length of hospital stay, while negatively correlated with lymphocyte counts and serum albumin. Moreover, strong correlations were observed between the frequency of CD14^+^HLA-DR^lo/neg^ MDSCs and T cell subsets, NK cell counts, and B cell percentages. The frequency of CD14^+^HLA-DR^lo/neg^ MDSCs could be used as a predictor of COVID-19 severity.

Conclusions: A high frequency of CD14^+^HLA-DR^lo/neg^ MDSCs, especially in severe patients, may indicate an immunoparalysis status and could be a predictor of disease severity and prognosis.

## INTRODUCTION

SARS-CoV-2 infection now has a global resonance, with the disease termed COVID-19. As of December 29, 2020, the most recent data confirm more than eighty-one million cases in 191 countries/regions, leading to over 1,770,000 deaths [1]. The illness severity of COVID-19 can range from mild to critical. Although most patients exhibit mild or moderate symptoms, it is expected that approximately 19% of patients will develop severe or critical disease, constituting a group with a high risk of death [2]. Some patients with mild symptoms in early stage will suddenly deteriorate to severe respiratory failure, requiring intubation and mechanical ventilation. These patients have a high risk of death, even reaching 60% mortality rate [3]. Hence, management of the disease progression of severe patients is essential for COVID-19 prevention and control. A large number of studies are currently concerned about the “cytokine storm” induced by COVID-19 [4]; however, over the course of severe illness, patients may show moderate to low fever, or even no obvious fever [5], and have dramatically reduced numbers of circulating T lymphocytes and NK cells [6], suggesting these patients may have developed immunosuppression similar to that seen in sepsis caused by bacteria [7]. When transitioning to an immunosuppressive state, monocytes are highly sensitive and become inactive, leading to a phase called "immunoparalysis" [8]. This is defined as a decrease in the level of HLA-DR expression on monocytes, measured by the frequency of one kind of myeloid-derived suppressor cells (MDSCs), defined as CD14^+^HLA-DR^lo/neg^ MDSCs [9]. MDSCs are a group of immature cells that can mediate suppression of the immune response. In mice, MDSCs are characterized as CD11b^+^Gr-1^+^Ly6G^+^ granulocyte MDSCs (G-MDSCs) and CD11b^+^Gr-1^+^Ly6C^+^ monocyte MDSCs (M-MDSCs) [10]. Nevertheless, the definition and role of G-MDSCs and M-MDSCs in human remain elusive due to the lack of specific phenotypic markers [11, 12]. Currently, G-MDSCs are mainly described as CD11b^+^CD33^+^CD15^+^HLA-DR^+^, whereas M-MDSCs are mainly defined as CD11b^+^CD33^+^CD14^+^HLA-DR^lo/neg^ or CD14^+^HLA-DR^lo/neg^ in human. Filipazzi P et al. identified and characterized for first time the new subset of MDSCs, CD14^+^HLA-DR^lo/neg^, in peripheral blood of melanoma patients with high suppressive of lymphocyte functions [13]. In clinical practice, we noticed that many COVID-19 patients admitted to ICU showed a significant decrease in T lymphocyte subsets, suggesting that they were in an immunosuppressive state. Therefore, we are interested in the difference between CD14^+^HLA-DR^lo/neg^ MDSC frequency in ICU patients and non-ICU patients, and the changes before and after negative SARS-CoV-2 nucleic acid tests.

## RESULTS

### Baseline characteristics

27 COVID-19 patients were included in our study. The median age was 58 years (IQR, 27-83). Of these, 17 (62.96%) were men and 10 (37.04%) were women (Table 1). Diabetes (14.81%) and hypertension (25.93%) were the two most common comorbidities in the patient group. Of the 27 cases, 8 patients (29.62%) were admitted to the ICU, and 19 patients were managed outside of the ICU (non-ICU). The levels of C-reactive protein, prothrombin time, lactate dehydrogenase and D-dimer were higher in ICU patients than non-ICU patients. Compared with non-ICU cases, the level of albumin was reduced in ICU patients (P=0.003). Moreover, duration of hospital stay was significantly longer for the ICU group compared to the non-ICU group (P=0.003).

**Table 1:** Clinical and laboratory characteristics of COVID-19 patients.

	**Total(n=27)**	**Non-ICU(n=19)**	**ICU(n=8)**	**P value**
age (IQR, y)	58(27-83)	56(27-80)	69.5(42-83)	0.062
male/female	17/10	12/7	5/3	0.774
main comorbidities (n, %)				
diabetes	4(14.81%)	2(10.53%)	2(25.00%)	0.558
hypertension	7(25.93%)	2(10.53%)	4(50.00%)^*^	*0.044*
length of hospitalization (IQR, d)	22(18-27)	20(17-23)	30(24-47)^*^	*0.003*
Laboratory values (IQR)				
leukocytes (×10^9^/L)	5.40(3.22-12.86)	4.77(3.22-12.86)	7.51(3.87-10.29)	0.111
neutrophils (×10^9^/L)	3.82(0.83-9.58)	3.57(0.83-9.46)	5.65(2.71-9.58)	0.100
lymphocyte (×10^9^/L)	1.00(0.23-2.11)	1.10(0.30-2.09)	0.71(0.23-2.11)	0.089
monocyte (×10^9^/L)	0.28(0.16-0.91)	0.28(0.19-0.91)	0.30(0.16-0.58)	0.832
C-reactive protein (mg/L)	10.10(2.52-22.00)	5.60(1.25-12.98)	43.90(11.50-81.19)^*^	*0.002*
procalcitonin (ng/ml)	0.03(0.02-0.05)	0.03(0.02-0.06)	0.04(0.02-0.06)	0.653
activated partial thromboplastin time (s)	23.75(20.58-23.75)	22.30(20.55-26.40)	30.65(21.25-35.90)	0.097
prothrombin time (s)	10.90(10.10-12.08)	10.60(10.10-11.20)	12.70(10.20-14.08)^*^	0.041
thrombin time (s)	17.80(16.33-21.15)	18.10(16.70-22.10)	17.10(15.20-20.75)	0.256
fibrinogen (g/L)	2.89(1.45-4.36)	2.89(1.45-3.87)	2.84(1.49-4.36)	0.473
D-Dimer(mg/L)	1.01(0.12-45.15)	0.53(0.12-15.55)	7.09(0.76-45.15)^*^	*0.002*
alanine transferase (U/L)	28.00(17.50-46.00)	28.00(16.50-43.50)	26.00(17.25-105.75)	*0.711*
aspartate aminotransferase (U/L)	25.00(17.50-41.00)	23.00(18.00-34.00)	34.00(15.50-60.00)	*0.366*
total protein (g/L)	62.60(58.65-67.55)	62.60(56.50-71.50)	62.75(60.00-63.78)	*0.977*
albumin (g/L)	39.10(32.90-49.00)	42.00(36.00-49.00)	36.80(32.90-39.10)^*^	*0.003*
creatinine (μmol/L)	57.00(47.25-75.00)	63.00(49.75-75.00)	49.30(39.05-38.95)	*0.307*
lactate dehydrogenase (U/L)	225.00(123.00-941.00)	211.00(123.00-397.00)	643.50(189.00-941.00)^*^	*0.011*
total bilirubin (μmol/L)	9.20(6.90-14.20)	9.90(7.88-15.83)	9.20(6.00-23.00)	*0.868*
creatine kinase (U/L)	62.00(34.00-106.00)	64.00(35.50-100.00)	72.50(36.75-176.00)	*0.555*

*, *vs* non-ICU, p<0.05. Abbreviations: IQR, interquartile range.

### Frequencies of CD14^+^HLA-DR^lo/neg^ MDSCs in ICU patients, non-ICU patients, and healthy controls

**Figure 1:**
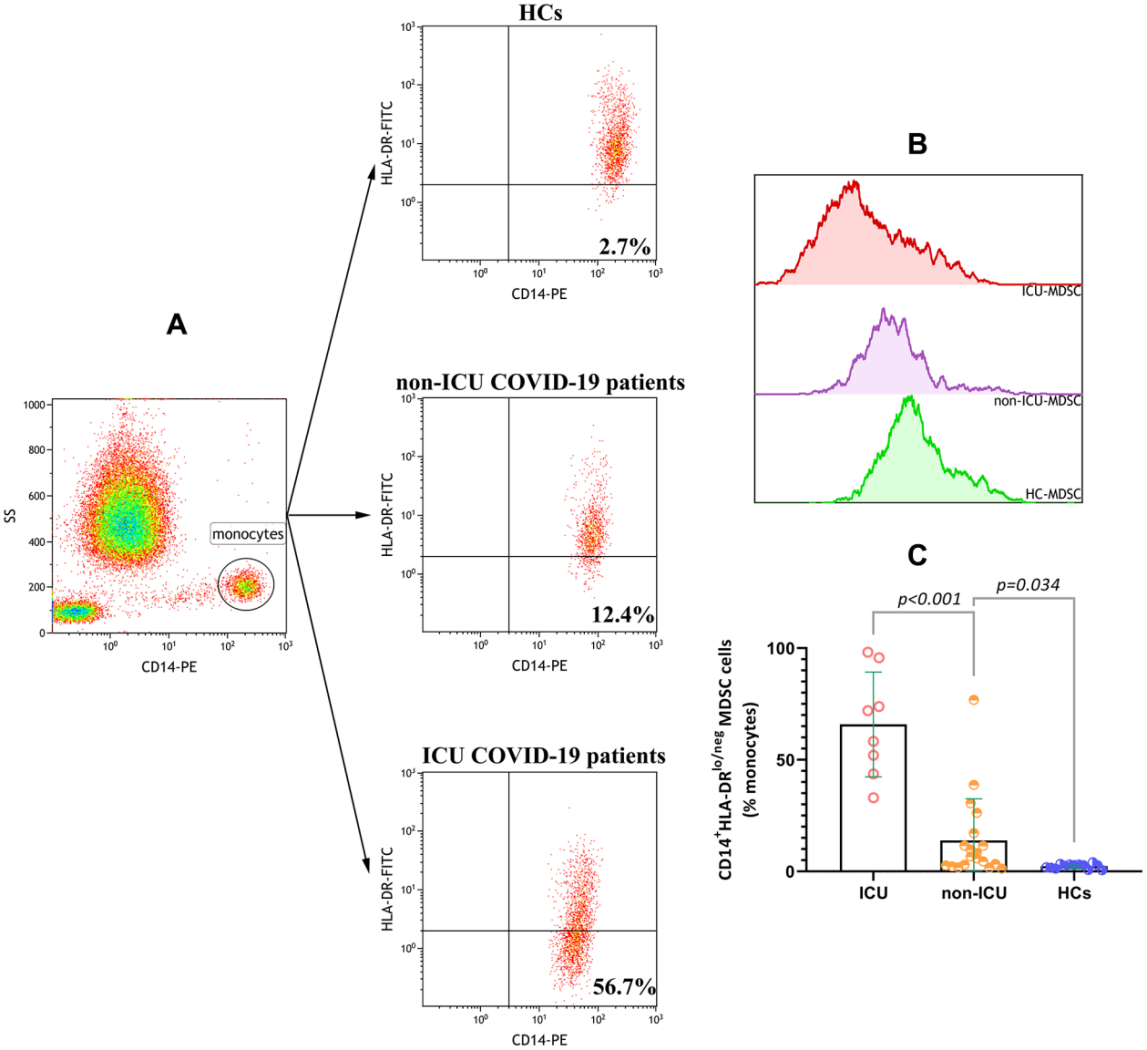
**Increased %CD14^+^HLA-DR^lo/neg^ MDSCs in COVID-19 patients.** (**A**) Representative gating strategy used to identify CD14^+^HLA-DR^lo/neg^ MDSCs in whole blood. (**B**) Flow cytometry overlay histograms for HLA-DR expressions on monocytes in HC, non-ICU COVID-19 patient and ICU COVID-19 patient. (**C**) Comparisons of CD14^+^HLA-DR^lo/neg^ MDSC(%) in HCs group, non-ICU group and ICU group. Abbreviations: ICU, intensive care unit; HCs, healthy controls; MDSC, myeloid-derived suppressor cells.

We determined the frequencies of CD14^+^HLA-DR^lo/neg^ MDSCs, characterized by low or negative expression of HLA-DR, in healthy controls (HCs) and COVID-19 patients. Peripheral CD14^+^HLA-DR^lo/neg^ MDSC frequencies were markedly increased in COVID-19 patients compared to HCs (P<0.0001). Furthermore, ICU patients had higher levels of CD14^+^HLA-DR^lo/neg^ MDSCs than non-ICU patients (P<0.001)(Figure 1).

### Changes in CD14^+^HLA-DR^lo/neg^ MDSCs before and after negative SARS-CoV-2 nucleic acid testing

**Figure 2:**
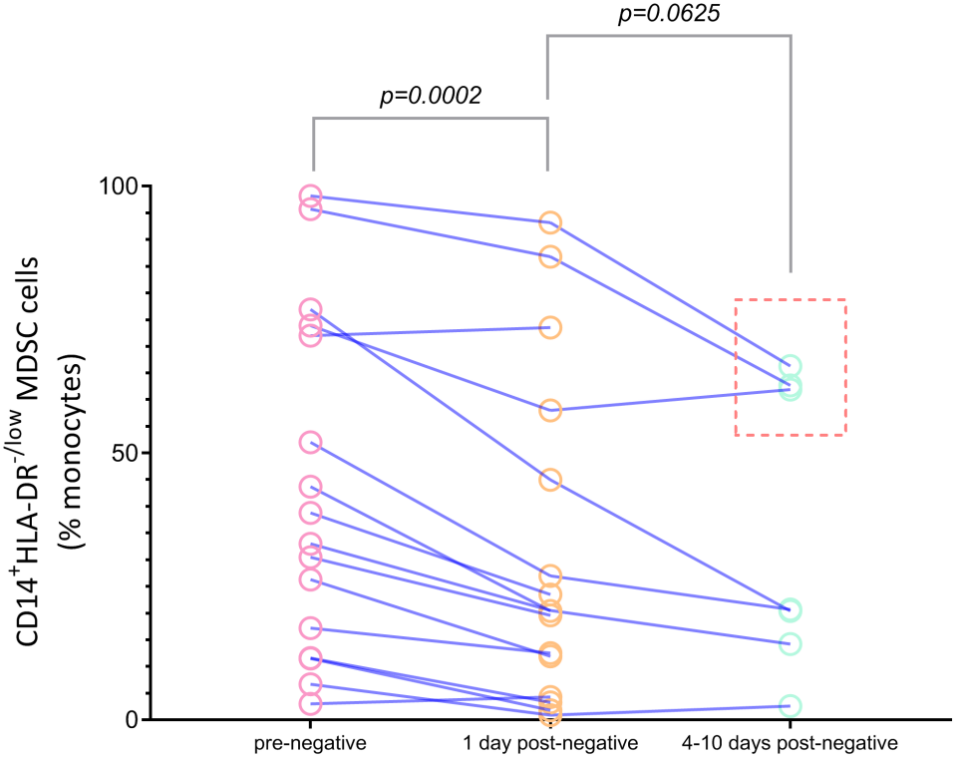
**Dynamic analysis of percentages of CD14^+^HLA-DR^lo/neg^ MDSCs in COVID-19 patients before (rose red), 1 day after (orange) and 4-10 days after (light green) virus nucleic acid negative conservation.** Abbreviations: MDSC, myeloid-derived suppressor cells.

**Figure 3:**
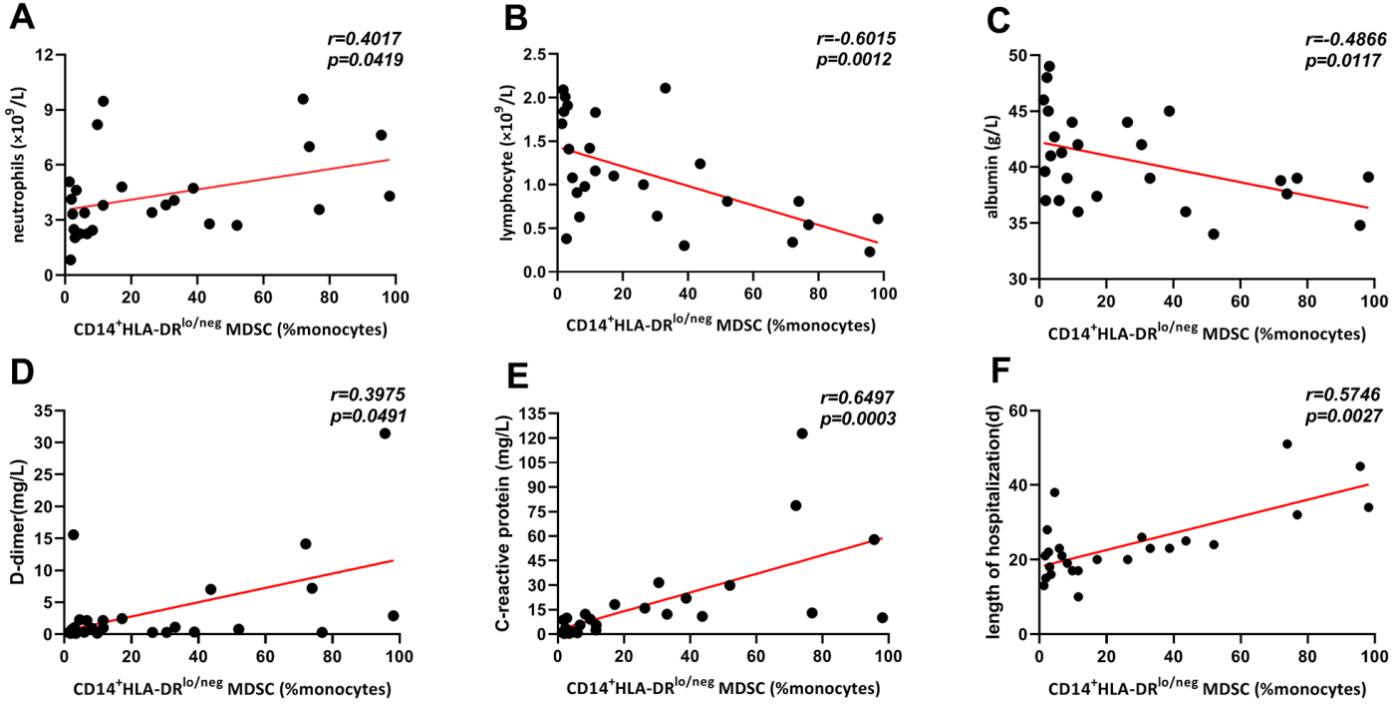
**Correlation of frequencies of CD14^+^HLA-DR^lo/neg^ MDSCs and clinical and laboratory indexes in all COVID-19 patients.** Frequencies of CD14^+^HLA-DR^lo/neg^ MDSCs were positively correlated with neutrophil (**A**), D-dimer (**D**), C-reactive protein (**E**) and length of hospitalization (**F**), and was negatively associated with lymphocyte (**B**) and albumin (**C**). Abbreviations: MDSCs, myeloid-derived suppressor cells.

To further investigate the clinical significance of CD14^+^HLA-DR^lo/neg^ MDSCs in COVID-19, we performed an evaluation of 15 COVID-19 patients (7 ICU patients and 8 non-ICU patients) before and 1 day after negative SARS-CoV-2 nucleic acid results, and in 7 patients (5 ICU patients and 2 non-ICU patients) before, 1 day after, and 4-10 days after testing negative for the virus. The data showed that there was a reduction in the levels of CD14^+^HLA-DR^lo/neg^ MDSCs 1 day after testing negative for SARS-CoV-2 when compared to the levels during active disease (P=0.0002) (Figure 2). Although the MDSC levels decreased 4-10 days after negative nucleic acid testing, the difference was not statistically significant (P>0.05). For 3 patients admitted to ICU, represented by the data points inside the dotted frame in Figure 3, high frequencies (>50.0%) of CD14^+^HLA-DR^lo/neg^ MDSCs were still observed 4-10 days after testing negative for viral nucleic acid.

### Elevated frequencies of CD14^+^HLA-DR^lo/neg^ MDSCs were correlated with neutrophil, lymphocyte, albumin, D-dimer, CRP, and length of hospitalization

The correlations between CD14^+^HLA-DR^lo/neg^ MDSCs frequencies and baseline clinical and laboratory indexes were determined and analyzed for COVID-19 patients. As shown in Figure 3, MDSCs frequencies were positively correlated with neutrophil count (weak), D-dimer (weak), C-reactive protein, and hospital stay, while inversely associated with lymphocyte count and serum albumin (weak). No obvious correlation was observed between MDSCs and other indicators.

### Increased frequencies of CD14^+^HLA-DR^lo/neg^ MDSCs were correlated with lymphocyte subsets

**Figure 4:**
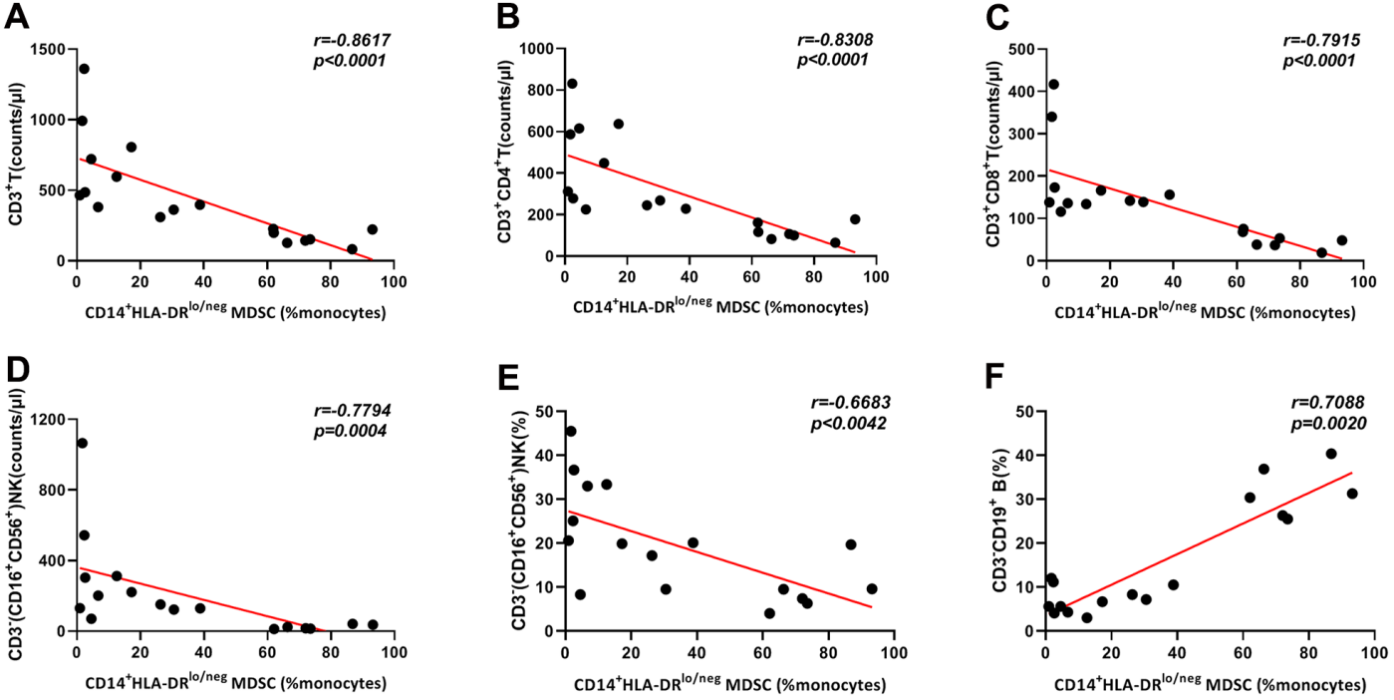
**Correlation of frequencies of CD14^+^HLA-DR^lo/neg^ MDSCs and lymphocyte subsets in 18 COVID-19 patients.** Frequencies of CD14^+^HLA-DR^lo/neg^ MDSCs showed negative correlations with CD3^+^T count (**A**), CD3^+^CD4^+^T count (**B**), CD3^+^CD8^+^T count (**C**), CD3^-^(CD16^+^CD56^+^)NK count (**D**) and CD3^-^(CD16^+^CD56^+^)NK percentage (**E**). CD14^+^HLA-DR^lo/neg^ MDSCs percentages were positively correlated with CD3^-^CD19^+^B cell frequencies (**F**). Abbreviations: MDSCs, myeloid-derived suppressor cells.

We obtained 18 data sets containing information on MDSCs and T cell subsets from COVID-19 patients, and the relationships between frequencies of CD14^+^HLA-DR^lo/neg^ MDSCs and lymphocyte subsets were analyzed. As shown in Figure 4, negative correlations were observed between the frequencies of MDSCs and the absolute count of CD3^+^, CD3^+^CD4^+^, and CD3^+^CD8^+^ T cells, and CD3^-^CD16^+^CD56^+^ NK cells in COVID-19 patients. In addition, we found that the frequency of MDSCs was negatively correlated with the percentage of CD3^-^CD16^+^CD56^+^ NK cells, but positively correlated with the frequency of CD3^-^CD19^+^ B cells. No correlation was found with frequencies of CD3^+^ (r=-0.1765, P=0.4836), CD3^+^CD4^+^(r=-0.0052, P=0.9838), and CD3^+^CD8^+^ T cells (r=-0.0939, P=0.7109), or the absolute count of CD3^-^CD19^+^ B cells (r=-0.1950, P=0.4505).

### Elevated frequency of CD14^+^HLA-DR^lo/neg^ MDSCs is correlated with SARS-CoV-2 viral RNA load

**Figure 5:**
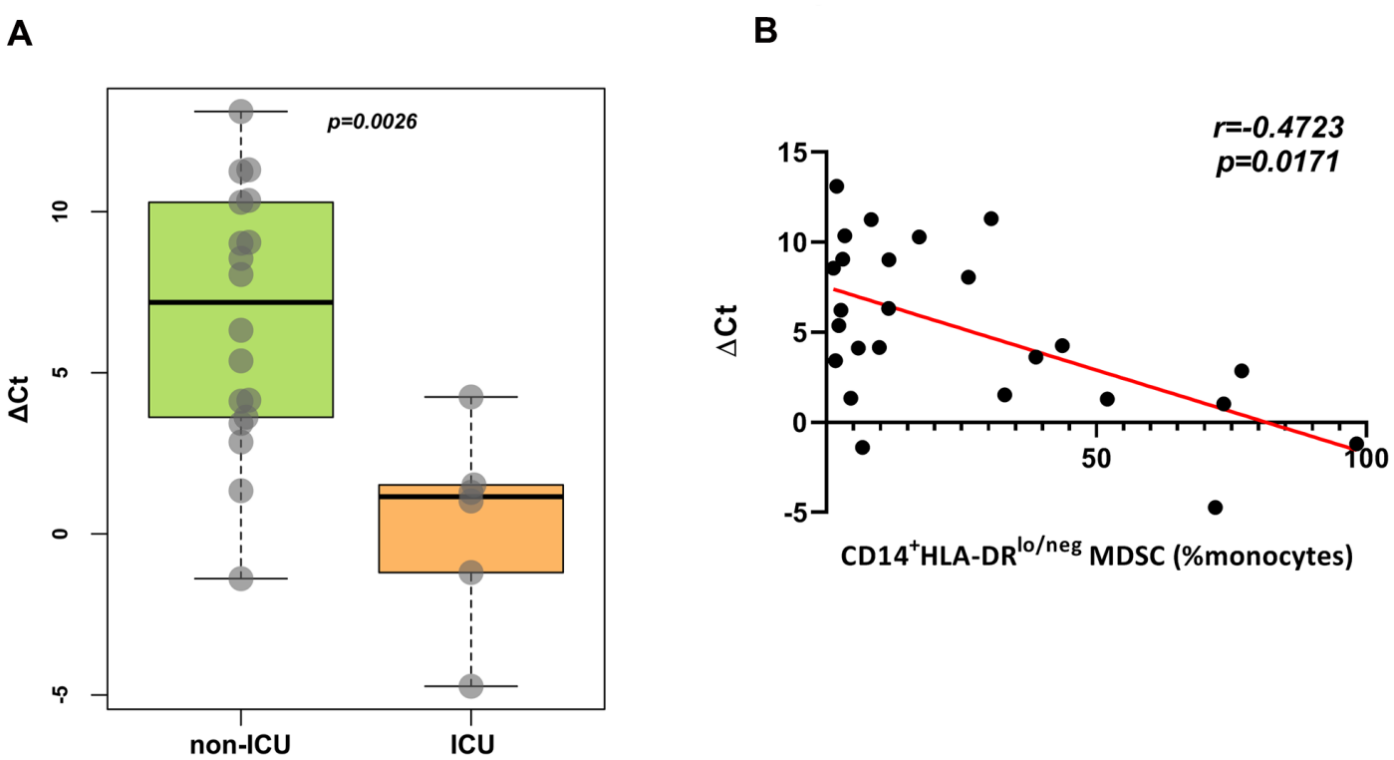
**Correlation of frequencies of CD14^+^HLA-DR^lo/neg^ MDSCs and viral loads.** (**A**), Viral loads were higher in ICU patients than non-ICU patients. (**B***)*, Frequencies of CD14^+^HLA-DR^lo/neg^ MDSCs were negatively correlated with ΔCt. Abbreviations: ICU, intensive care unit; MDSC, myeloid-derived suppressor cells; Ct, cycle threshold.

We analyzed the relationship between frequencies of CD14^+^HLA-DR^lo/neg^ MDSCs and viral RNA loads, and the results indicated patients with COVID-19 with more severe disease requiring ICU admission had high viral RNA loads when compared to non-ICU patients. And a negative correlation (weak, r=-0.4723) was observed between CD14^+^HLA-DR^lo/neg^ MDSCs percentages and viral loads (Figure 5).

## DISCUSSION

The treatment of severe COVID-19 patients has been a focus in the prevention and management of SARS-CoV-2 infection due to the higher mortality [14]. However, little is known about the detailed factors governing the development or remission of severe disease. To date, the most important predictors of COVID-19 severity were related to the activation or suppression of the host's immune response [7]. Immune system dysregulation has been observed and is believed to be related to the severity of COVID-19 [15]. Zheng et al. recently discovered that increased levels of T cell exhaustion and reduced diversity of T cell function may be indicators of disease progression in COVID-19 patients [16]. Similarly, our previous study found severe COVID-19 patients had significantly reduced lymphocyte subset counts compared to mild-to-moderate patients [17]. However, the mechanism underlying T cell lymphopenia remains unknown. High levels of anti-inflammatory mediators, such as IL-10 and IL-4, have also been reported in COVID-19 patients, particularly ICU patients [18]. These results suggest that dysregulation of the immune response may present in the form of immunosuppression as well as a cytokine storm. Indeed, it has been reported that COVID-19 is associated with states of both immunodeficiency and hyperinflammation [19]. Furthermore, even without significant hypotension, many severe or critically ill COVID-19 patients develop typical clinical manifestations of shock. Severe metabolic acidosis and impaired liver and kidney function caused by SARS-CoV-2 infection indicates that viral sepsis may occur in severe patients [20]. The low expression of HLA-DR on monocytes in critically ill patients is the most widely recognized sign of innate immune suppression, and the most studied biomarker of sepsis-induced immune suppression, termed “immunoparalysis” [21]. Monocytes with low or no HLA-DR expression are often referred to as CD14^+^HLA-DR^lo/neg^ MDSCs. HLA-DR is an MHC class II glycoprotein expressed on antigen presenting cells, which presents peptides derived from antigens to T cell receptors, resulting in T cell activation [9, 22]. Therefore, the ability of CD14^+^HLA-DR^lo/neg^ MDSCs to present antigens to T cells is diminished, and many previous studies have demonstrated that MDSCs have a strong immunosuppressive effect [23]. Volk et al. defined immunoparalysis in sepsis as CD14^+^HLA-DR^lo/neg^ MDSCs being >70% of the monocyte pool [8]. Studies have shown that the decrease of HLA-DR expression on monocytes is a vital predictor of forthcoming sepsis [24]. In this study, we found that COVID-19 patients showed significant increases in the frequency of CD14^+^HLA-DR^lo/neg^ MDSCs when compared to HCs, especially in patients admitted to ICU. This may signify immunosuppression in severe COVID-19 patients, and also suggests that while focusing on the treatment of cytokine storms clinically, we should also pay attention to possible immunosuppression in patients to prevent secondary infection. Using single-cell RNA sequencing and single-cell proteomics of whole blood and peripheral blood mononuclear cells, Schulte-Schrepping J et al. identified a high proportion of HLA-DR^lo^ monocytes clusters in severe COVID-19 patients, and low expression of HLA-DR is an established marker of monocyte dysfunction as well as immunosuppression in sepsis [25]. Furthermore, monocytes isolated from COVID-19 patients exhibited impaired cytokine secretion upon LPS stimulation, particularly severe cases, indicating an immunosuppressive phenotype of monocytes in COVID-19 patients. This is consistent with the results of the present study. In addition, they also found that HLA-DR^hi^ monocytes were predominant in mild patients, while HLA-DR^lo^ monocytes were present mainly in severe patients, with HLA-DR^lo^CD163^hi^ monocytes in the early stage and HLA-DR^lo^S100A^hi^ monocytes at the later stage. The present study found frequencies of HLA-DR^hi^ monocytes were still relatively high after testing negative for SARS-CoV-2 nucleic acid. Unfortunately, what is not clear is which kind of HLA-DR^lo^ monocytes is the main form at this phase. In addition, elucidating the underlying mechanism of the increased frequency of MDSCs in COVID-19 patients requires further study. High CD14^+^HLA-DR^lo/neg^ MDSC frequencies have also been reported in other patients with viral infection, such as hepatitis B [26] and hepatitis C [27]. In patients with H7N9 infection, Diao et al. found that the expression of HLA-DR on CD14^+^ cells was significantly lower in patients with severe than mild disease and, importantly, was related to the severity of H7N9 infection [28].

According to “Notice on printing and distributing the diagnosis and treatment plan of pneumonia with new coronavirus infection (trial version 7)”, two consecutive negative RT-PCR test results at least 24 h apart is one of the criteria for patient discharge, which to some extent indicates recovery from infection. We found that the frequency of MDSCs decreased significantly 1 day after negative test results for SARS-CoV-2 compared to the frequency during active disease, suggesting that level of CD14^+^HLA-DR^lo/neg^ MDSCs was related to viral infection. Subsequent follow-up analysis found that circulating CD14^+^HLA-DR^lo/neg^ MDSC frequency was significantly lower post-infection compared to the levels observed during infection, however it was still significantly higher 4-10 days post-infection for severe patients compared to HCs. High frequencies were observed in 3 ICU patients even 4-10 days after negative viral nucleic acid results, indicating that patients with severe COVID-19 may still have abnormal immunity after testing negative for SARS-CoV-2, and these patients may require further monitoring treatment. Agrati C et al. demonstrated high levels of another MDSCs, G-MDSCs, in COVID-19 patients, as high as 95% in severe patients, which could significantly suppress T-cell activation; the frequencies of G-MDSCs decreasing with recovery [29]. This is consistent with the trend of M-MDSCs in the present study, suggesting that both MDSCs may be involved in the immune abnormal process in COVID-19 patients. In addition, for patients with severe disease, G-MDSCs decreased in the later stage, yet were still remained higher level compared with those in healthy population. The current dynamic follow-up analysis also revealed that the proportion of M-MDSCs was higher than in healthy subjects even 4-10 days post-infection. These results suggested that persistent abnormal MDSCs, including G-MDSCs and M-MDSCs, may be accompanied by a persistent state of “immunoparalysis”. Chen et al. reported that in severe H7N9 patients, the total lymphocyte count and percentages of different lymphocyte subtypes returned to normal levels 1 month post-infection, while 3 months were needed for the normalization of CD14^+^HLA-DR^lo/neg^ MDSCs [30]. Unfortunately, our study did not record when the CD14^+^HLA-DR^lo/neg^ MDSC frequency of COVID-19 patients returned to normal levels.

Lymphopenia is a common sign of COVID-19 that has been observed in more than 70% of patients, particularly severe patients [31, 32]; however the mechanism underlying this observation remains unclear. Interestingly, the frequency of CD14^+^HLA-DR^lo/neg^ MDSCs showed negative correlation to lymphocyte counts, suggesting that MDSCs may be involved in the lymphopenia detected in COVID-19. The relationship between malnutrition and infections is a vicious cycle, whereby not only are the risk and severity of infections increased by malnutrition, but malnutrition may also result from infection [33]. During infection induced-hyperinflammation, serum albumin is reduced. Previous studies have shown that hypoalbuminemia can indicate a high risk of death for patients with sepsis [34]. We also found a notable decrease of albumin and increase of CRP in COVID-19 patients, especially in ICU patients, which is consistent with a recent study [35]. The correlations between MDSCs and albumin and CRP imply that a high MDSC percentage may be an indicator of the systematic inflammatory and nutritional state, and the significant correlation between CD14^+^HLA-DR^lo/neg^ MDSC percentage and length of hospital stay implies that this may be a prognostic predictor for COVID-19 patients.

Studies have reported that the number of total T cells, and CD4^+^ and CD8^+^ T cells, were dramatically reduced in COVID-19 patients, especially in patients requiring ICU care, and this was negatively correlated with patient survival [36]. It is well established that CD14^+^HLA-DR^lo/neg^ MDSCs mediate immunosuppression in tumors by suppressing the proliferation of T cells [37, 38]. Thus, what role does the expanded MDSC population play in COVID-19? We found that MDSC frequency showed a strong inverse correlation with counts of T cell subsets and NK cells. These results indicate that the decrease in lymphocyte subsets in COVID-19 may be partly due to the expansion of MDSCs in response to hyperinflammation. Further research needs to be carried out to explore the underlying mechanism of this observation. Moreover, a positive correlation was found between CD14^+^HLA-DR^lo/neg^ MDSCs frequencies and SARS-CoV-2 viral RNA loads, indicating SARS-CoV-2 infection may cause immunosuppression through inducing expansion of CD14^+^HLA-DR^lo/neg^ MDSCs. Indeed, high levels of MDSCs were found in patients experiencing viral infections, such as dengue, and correlated with virus viral load and severity [39].

The limitations of our study include the lack of longer follow-up and the relatively low number of patients involved. The COVID-19 situation in China has been effectively controlled, and there are no new-onset cases in the local area. We cannot expand the study population for further research, and we suggest dynamic monitor of CD14^+^HLA-DR^lo/neg^ MDSCs in COVID-19 patients could help to evaluate its detailed clinical values and roles in development of COVID-19 in current outbreak areas.

In conclusion, we observed a high frequency of immunosuppressive CD14^+^HLA-DR^lo/neg^ MDSCs in COVID-19 patients, particularly ICU patients. Patients with high percentages of CD14^+^HLA-DR^lo/neg^ MDSCs seemed to have lower ALB, lower T cell and NK cell counts, and higher levels of inflammation and viral loads, and required a longer hospital stay. Moreover, circulating CD14^+^HLA-DR^lo/neg^ MDSC percentages may be a predictor of disease severity. These results suggest that more urgent early intervention may be required for patients with high CD14^+^HLA-DR^lo/neg^ MDSC percentages. Furthermore, high frequencies of MDSCs observed even 4-10 days after negative SARS-CoV-2 nucleic acid testing suggest that after severe infection, the recovery of the antigen-presenting capability of monocytes, and thus normal immune function, was delayed. This may lead to increased vulnerability to secondary infections. These findings may help elucidate the pathogenesis of COVID-19 and develop novel biomarkers and therapeutic strategies.

## MATERIALS AND METHODS

### Subjects

From February 10 to March 15, 2020, 27 patients with COVID-19, confirmed by the detection of SARS-CoV-2 RNA in nasopharyngeal swab samples and diagnosed and classified according to the Guidelines for diagnosis and management of COVID-19 (7th edition, in Chinese) issued by the National Health Commission of China [40], were enrolled in our study. After two consecutive negative results for nucleic acid in respiratory samples separated by at least 24 hours, a patient was considered negative for SARS-CoV-2. Of these 27 cases, 8 patients (29.62%) had severe or critically ill COVID-19 and were admitted to the ICU, and 19 patients who were mild to moderate were not admitted to the ICU. Adults with severe disease were classified based on the following conditions: (1) dyspnea with respiratory rate (RR) ≥ 30 breaths/minute; (2) resting state with oxygen saturation ≤ 93%; (3) arterial oxygen partial pressure (PaO_2_)/oxygen concentration (FiO_2_) ≤ 300 mmHg; (4) lung imaging showing significant > 50% progression of the disease within 24-48 hours. The patient is considered critically ill if any of the following criteria are met. (1) respiratory failure requiring mechanical ventilation; (2) shock; and (3) combined organ failure and ICU monitoring. In addition, a total of 13 age- and gender- matched healthy subjects were included as a control group. This study was approved by the Ethics Commission of the First Affiliated Hospital of Nanchang University and was performed in accordance with the Declaration of Helsinki. Written informed consent was obtained from all participants.

### Data collection

Demographic information and comorbidities were collected. Blood cells count, biochemical indexes, coagulation parameters and inflammatory marker were also collected. The thresholds selected for laboratory measurements were based on the normal range set by each hospital. The viral loads from nasopharyngeal were estimated by the ΔCt method (Ct_sample_- Ct_ref_).

### Flow cytometry

EDTA-anticoagulated peripheral blood samples were collected to assess lymphocyte subsets and CD14^+^HLA-DR^lo/neg^ MDSCs. The following antibodies (Beckman Coulter) were used for flow cytometry analysis: CD45-FITC/CD4-PE/CD8-ECD/CD3-PC5 for detection of T lymphocyte subsets; CD3-FITC/CD(16+56)-PE for detection of NK cells; CD3-ECD and CD19-PC5 for detection of B cells; CD14-PE and HLA-DR-FITC for detection of MDSCs. Flow cytometry was performed using FC-500 (Beckman Coulter, USA). Data analysis was carried out with Kaluza Analysis 2.1 Flow Software.

### Statistical analysis

Statistical analyses were performed using SPSS 23.0 and GraphPad Prism 8.0. Categorical variables were described using percentages, and continuous variables were described as mean±SD or median (interquartile range, IQR). When the data were normally distributed, the means of continuous variables were compared using the independent t-test; otherwise, the Mann-Whitney U test was used. Categorical variables were compared using the χ^2^ test. Likewise, the Pearson method was used for correlation analysis. A value of P <0.05 was considered statistically significant.

## References

[1] Coronavirus 2019-nCoV, CSSE. Coronavirus 2019-nCoV Global Cases by Johns Hopkins CSSE. https://coronavirus.jhu.edu/map.html. 2020.

[2] Wu Z, McGoogan JM. Characteristics of and important lessons from the coronavirus disease 2019 (COVID-19) outbreak in China: summary of a report of 72 314 cases from the Chinese center for disease control and prevention. JAMA. 2020; 323:1239–42. 10.1001/jama.2020.2648 32091533

[3] Arabi YM, Murthy S, Webb S. COVID-19: a novel coronavirus and a novel challenge for critical care. Intensive Care Med. 2020; 46:833–36. 10.1007/s00134-020-05955-1 32125458 PMC7080134

[4] Vaninov N. In the eye of the COVID-19 cytokine storm. Nat Rev Immunol. 2020; 20:277. 10.1038/s41577-020-0305-6 32249847 PMC7132547

[5] Guan WJ, Ni ZY, Hu Y, Liang WH, Ou CQ, He JX, Liu L, Shan H, Lei CL, Hui DS, Du B, Li LJ, Zeng G, et al, and China Medical Treatment Expert Group for Covid-19. Clinical characteristics of coronavirus disease 2019 in China. N Engl J Med. 2020; 382:1708–20. 10.1056/NEJMoa2002032 32109013 PMC7092819

[6] Wang F, Nie J, Wang H, Zhao Q, Xiong Y, Deng L, Song S, Ma Z, Mo P, Zhang Y. Characteristics of peripheral lymphocyte subset alteration in COVID-19 pneumonia. J Infect Dis. 2020; 221:1762–69. 10.1093/infdis/jiaa150 32227123 PMC7184346

[7] Vardhana SA, Wolchok JD. The many faces of the anti-COVID immune response. J Exp Med. 2020; 217:e20200678. 10.1084/jem.20200678 32353870 PMC7191310

[8] Volk HD, Reinke P, Krausch D, Zuckermann H, Asadullah K, Müller JM, Döcke WD, Kox WJ. Monocyte deactivation—rationale for a new therapeutic strategy in sepsis. Intensive Care Med. 1996 (Suppl 4); 22:S474–81. 10.1007/BF01743727 8923092

[9] Mengos AE, Gastineau DA, Gustafson MP. The CD14^+^ HLA-DR^lo/neg^ monocyte: an immunosuppressive phenotype that restrains responses to cancer immunotherapy. Front Immunol. 2019; 10:1147. 10.3389/fimmu.2019.01147 31191529 PMC6540944

[10] Gabrilovich DI, Nagaraj S. Myeloid-derived suppressor cells as regulators of the immune system. Nat Rev Immunol. 2009; 9:162–74. 10.1038/nri2506 19197294 PMC2828349

[11] Manjili MH. Phenotypic plasticity of MDSC in cancers. Immunol Invest. 2012; 41:711–21. 10.3109/08820139.2012.673670 23017142

[12] Zhao F, Hoechst B, Duffy A, Gamrekelashvili J, Fioravanti S, Manns MP, Greten TF, Korangy F. S100A9 a new marker for monocytic human myeloid-derived suppressor cells. Immunology. 2012; 136:176–83. 10.1111/j.1365-2567.2012.03566.x 22304731 PMC3403264

[13] Filipazzi P, Valenti R, Huber V, Pilla L, Canese P, Iero M, Castelli C, Mariani L, Parmiani G, Rivoltini L. Identification of a new subset of myeloid suppressor cells in peripheral blood of melanoma patients with modulation by a granulocyte-macrophage colony-stimulation factor-based antitumor vaccine. J Clin Oncol. 2007; 25:2546–53. 10.1200/JCO.2006.08.5829 17577033

[14] Li H, Liu SM, Yu XH, Tang SL, Tang CK. Coronavirus disease 2019 (COVID-19): current status and future perspectives. Int J Antimicrob Agents. 2020; 55:105951. 10.1016/j.ijantimicag.2020.105951 32234466 PMC7139247

[15] Wen W, Su W, Tang H, Le W, Zhang X, Zheng Y, Liu X, Xie L, Li J, Ye J, Dong L, Cui X, Miao Y, et al. Immune cell profiling of COVID-19 patients in the recovery stage by single-cell sequencing. Cell Discov. 2020; 6:31. 10.1038/s41421-020-0168-9 32377375 PMC7197635

[16] Zheng HY, Zhang M, Yang CX, Zhang N, Wang XC, Yang XP, Dong XQ, Zheng YT. Elevated exhaustion levels and reduced functional diversity of T cells in peripheral blood may predict severe progression in COVID-19 patients. Cell Mol Immunol. 2020; 17:541–43. 10.1038/s41423-020-0401-3 32203186 PMC7091621

[17] Jiang M, Guo Y, Luo Q, Huang Z, Zhao R, Liu S, Le A, Li J, Wan L. T-cell subset counts in peripheral blood can be used as discriminatory biomarkers for diagnosis and severity prediction of coronavirus disease 2019. J Infect Dis. 2020; 222:198–202. 10.1093/infdis/jiaa252 32379887 PMC7239156

[18] Huang C, Wang Y, Li X, Ren L, Zhao J, Hu Y, Zhang L, Fan G, Xu J, Gu X, Cheng Z, Yu T, Xia J, et al. Clinical features of patients infected with 2019 novel coronavirus in Wuhan, China. Lancet. 2020; 395:497–506. 10.1016/S0140-6736(20)30183-5 31986264 PMC7159299

[19] Jamilloux Y, Henry T, Belot A, Viel S, Fauter M, El Jammal T, Walzer T, François B, Sève P. Should we stimulate or suppress immune responses in COVID-19? Cytokine and anti-cytokine interventions. Autoimmun Rev. 2020; 19:102567. 10.1016/j.autrev.2020.102567 32376392 PMC7196557

[20] Li H, Liu L, Zhang D, Xu J, Dai H, Tang N, Su X, Cao B. SARS-CoV-2 and viral sepsis: observations and hypotheses. Lancet. 2020; 395:1517–20. 10.1016/S0140-6736(20)30920-X 32311318 PMC7164875

[21] Leijte GP, Rimmelé T, Kox M, Bruse N, Monard C, Gossez M, Monneret G, Pickkers P, Venet F. Monocytic HLA-DR expression kinetics in septic shock patients with different pathogens, sites of infection and adverse outcomes. Crit Care. 2020; 24:110. 10.1186/s13054-020-2830-x 32192532 PMC7082984

[22] Veglia F, Perego M, Gabrilovich D. Myeloid-derived suppressor cells coming of age. Nat Immunol. 2018; 19:108–19. 10.1038/s41590-017-0022-x 29348500 PMC5854158

[23] Laborde RR, Lin Y, Gustafson MP, Bulur PA, Dietz AB. Cancer vaccines in the world of immune suppressive monocytes (CD14(+)HLA-DR(lo/neg) cells): the gateway to improved responses. Front Immunol. 2014; 5:147. 10.3389/fimmu.2014.00147 24772111 PMC3983500

[24] Zhang DP, Yan FL, Xu HQ, Zhu YX, Yin Y, Lu HQ. A decrease of human leucocyte antigen-DR expression on monocytes in peripheral blood predicts stroke-associated infection in critically-ill patients with acute stroke. Eur J Neurol. 2009; 16:498–505. 10.1111/j.1468-1331.2008.02512.x 19187263

[25] Schulte-Schrepping J, Reusch N, Paclik D, Baßler K, Schlickeiser S, Zhang B, Krämer B, Krammer T, Brumhard S, Bonaguro L, De Domenico E, Wendisch D, Grasshoff M, et al, and Deutsche COVID-19 OMICS Initiative (DeCOI). Severe COVID-19 is marked by a dysregulated myeloid cell compartment. Cell. 2020; 182:1419–40.e23. 10.1016/j.cell.2020.08.001 32810438 PMC7405822

[26] Huang A, Zhang B, Yan W, Wang B, Wei H, Zhang F, Wu L, Fan K, Guo Y. Myeloid-derived suppressor cells regulate immune response in patients with chronic hepatitis B virus infection through PD-1-induced IL-10. J Immunol. 2014; 193:5461–69. 10.4049/jimmunol.1400849 25344470

[27] Ning G, She L, Lu L, Liu Y, Zeng Y, Yan Y, Lin C. Analysis of monocytic and granulocytic myeloid-derived suppressor cells subsets in patients with hepatitis C virus infection and their clinical significance. Biomed Res Int. 2015; 2015:385378. 10.1155/2015/385378 25815313 PMC4359884

[28] Diao H, Cui G, Wei Y, Chen J, Zuo J, Cao H, Chen Y, Yao H, Tian Z, Li L. Severe H7N9 infection is associated with decreased antigen-presenting capacity of CD14+ cells. PLoS One. 2014; 9:e92823. 10.1371/journal.pone.0092823 24664315 PMC3963940

[29] Agrati C, Sacchi A, Bordoni V, Cimini E, Notari S, Grassi G, Casetti R, Tartaglia E, Lalle E, D’Abramo A, Castilletti C, Marchioni L, Shi Y, et al. Expansion of myeloid-derived suppressor cells in patients with severe coronavirus disease (COVID-19). Cell Death Differ. 2020; 27:3196–207. 10.1038/s41418-020-0572-6 32514047 PMC7278239

[30] Chen J, Cui G, Lu C, Ding Y, Gao H, Zhu Y, Wei Y, Wang L, Uede T, Li L, Diao H. Severe infection with avian influenza a virus is associated with delayed immune recovery in survivors. Medicine (Baltimore). 2016; 95:e2606. 10.1097/MD.0000000000002606 26844470 PMC4748887

[31] Wang D, Hu B, Hu C, Zhu F, Liu X, Zhang J, Wang B, Xiang H, Cheng Z, Xiong Y, Zhao Y, Li Y, Wang X, Peng Z. Clinical characteristics of 138 hospitalized patients with 2019 novel coronavirus-infected pneumonia in Wuhan, China. JAMA. 2020; 323:1061–69. 10.1001/jama.2020.1585 32031570 PMC7042881

[32] Lovato A, de Filippis C. Clinical presentation of COVID-19: a systematic review focusing on upper airway symptoms. Ear Nose Throat J. 2020; 99:569–76. 10.1177/0145561320920762 32283980

[33] Katona P, Katona-Apte J. The interaction between nutrition and infection. Clin Infect Dis. 2008; 46:1582–88. 10.1086/587658 18419494

[34] Takegawa R, Kabata D, Shimizu K, Hisano S, Ogura H, Shintani A, Shimazu T. Serum albumin as a risk factor for death in patients with prolonged sepsis: an observational study. J Crit Care. 2019; 51:139–44. 10.1016/j.jcrc.2019.02.004 30825787

[35] Liu Y, Yang Y, Zhang C, Huang F, Wang F, Yuan J, Wang Z, Li J, Li J, Feng C, Zhang Z, Wang L, Peng L, et al. Clinical and biochemical indexes from 2019-nCoV infected patients linked to viral loads and lung injury. Sci China Life Sci. 2020; 63:364–74. 10.1007/s11427-020-1643-8 32048163 PMC7088566

[36] Liu Z, Long W, Tu M, Chen S, Huang Y, Wang S, Zhou W, Chen D, Zhou L, Wang M, Wu M, Huang Q, Xu H, et al. Lymphocyte subset (CD4+, CD8+) counts reflect the severity of infection and predict the clinical outcomes in patients with COVID-19. J Infect. 2020; 81:318–56. 10.1016/j.jinf.2020.03.054 32283159 PMC7151318

[37] Huang H, Zhang G, Li G, Ma H, Zhang X. Circulating CD14(+)HLA-DR(-/low) myeloid-derived suppressor cell is an indicator of poor prognosis in patients with ESCC. Tumour Biol. 2015; 36:7987–96. 10.1007/s13277-015-3426-y 25967454

[38] Huang A, Zhang B, Wang B, Zhang F, Fan KX, Guo YJ. Increased CD14(+)HLA-DR (-/low) myeloid-derived suppressor cells correlate with extrathoracic metastasis and poor response to chemotherapy in non-small cell lung cancer patients. Cancer Immunol Immunother. 2013; 62:1439–51. 10.1007/s00262-013-1450-6 23760662 PMC11028777

[39] Guo PL, Li LH, Li WL, Zhao JC, Hu FY, Zhang FC, Cai WP, Tang XP. The clinical significance of myeloid-derived suppressor cells in dengue fever patients. BMC Infect Dis. 2019; 19:926. 10.1186/s12879-019-4574-2 31675923 PMC6824033

[40] National Health Commission & National Administration of Traditional Chinese Medicine on March 3, 2020. Diagnosis and Treatment Protocol for Novel Coronavirus Pneumonia (Trial Version 7). Chin Med J (Engl). 2020; 133:1087–95. 10.1097/CM9.0000000000000819 32358325 PMC7213636

